# A Proposal for Research Involving New Biomarkers of Hypertension, Lifestyle, and Environmental Exposure

**DOI:** 10.3390/cimb47030206

**Published:** 2025-03-18

**Authors:** Angelika Edyta Charkiewicz

**Affiliations:** Department of Clinical Molecular Biology, Medical University of Bialystok, 15-269 Białystok, Poland; angelika.charkiewicz@umb.edu.pl

**Keywords:** lifestyle, new biomarkers, hypertension, early intervention, prevention

## Abstract

The constant monitoring of the population’s diet and assessment of occupational exposure and environmental impacts are the key to determining health risks and understanding the factors contributing to potential abnormalities in developing lifestyle diseases. Extensive long-term lifestyle monitoring studies can provide data on population health risks, including the most common cardiovascular diseases like hypertension. This paper presents research recommendations for future researchers and doctors to improve the diagnosis of hypertension and targeted, personalised treatment. The research proposal includes a lifestyle study, a diagnostic panel with new biomarkers, and an environmental exposure assessment of men working in the metallurgical industry. New developments and improved interventions are constantly being sought, including new biomarkers with high diagnostic utility for cardiovascular diseases like hypertension. This should enable early diagnosis, and consequently allow for appropriate and, most importantly, personalised therapy, and prevent an increase in CVD deaths. Only the effective diagnosis, treatment, and monitoring of hypertension can reduce the risk of developing diseases associated with hypertension. I propose that several new parameters (NO, cfDNA, MPO, PCSK9, MyBPC3, microRNA, TAS, Pb, and Cd) with prognostic and/or predictive potential should be included in screening to confirm the need for the extensive testing of middle-aged men by healthcare professionals due to the risk of hypertension.

## 1. Introduction

Cardiovascular diseases (CVDs) are the most common cause of mortality and incidence in Poland. According to a population forecast by Statistics Poland, the number of CVD-related deaths in Poland will peak at 223,300 per year in 2045, accounting for more than 50% of all projected deaths. In 2021, the number of CVD deaths in Poland stood at 180,760, or 473.7 per 100,000 population [1], while in Eastern Europe, this figure was 432.3 per 100,000 in 2022 [2]. The standardised death rate due to CVDs in 2021 in Poland for men was 648.1 and, thus, 47.3% higher than for women, whose rate was 440.1 [1]. This indicates significant differences in CVD prevalence between the sexes. After eliminating differences in age structure, it appears that CVDs represent a significantly higher risk in men.

The most common cardiovascular diseases are myocardial infarction, stroke, hypertension, atherosclerosis, or venous insufficiency, which are by far the most life-threatening. The main risk factors for the most damaging cardiovascular diseases (CVDs), such as myocardial infarction and stroke, include hypertension, smoking, abdominal obesity (particularly dangerous in men), alcohol abuse, diabetes, physical inactivity, unhealthy diet, dyslipidaemia, and psychosocial factors [3,4]. Lifestyle, especially diet, is one of the direct risk factors for cardiovascular diseases [2,5].

Too few studies involving long-term population monitoring and a lack of long-term analyses of the same study group can result in underestimations and prevent accounting for changing lifestyles, including consumption trends and ageing [6,7]. The constant monitoring of the population’s diet and assessment of occupational exposure and environmental impact play a key role in identifying health risks and understanding the factors contributing to potential abnormalities related to CVD progression [6,8,9,10,11].

Nutrients, vitamins, and minerals should be considered when assessing lifestyle in general, including diet. For example, an excess of copper (Cu) in the diet damages the cardiac muscle and coronary vessels, while copper deficiency can lead to cholesterol metabolism disorders [12,13]. In contrast, the margin between the required and toxic levels for selenium (Se) is very narrow [8,14]. Selenium is an antioxidant and protects against the harmful effects of heavy metals. It shows a high affinity for cadmium (Cd) and forms a complex with lead (Pb), effectively reducing their plasma levels [15]. Men working in the metallurgical industry worldwide are exposed to heavy metals in the workplace, which is not without consequences for their health. These metals enter their bodies through two main routes of exposure: oral and inhalation (atsdr.cdc.gov) [9,10,11,16,17]. Once absorbed by the body, heavy metals accumulate in the blood and bones, as well as in the liver, kidneys, brain, and skin. Their acute and chronic adverse health effects depend on how fast they are eliminated [7,9,18,19,20,21,22]. Furthermore, human exposure to heavy metals increases with environmental pollution [2,9,10,22,23]. An imbalance of these and other heavy metals promotes cardiovascular diseases by disrupting the body’s oxidative–antioxidative balance [8,10,11,22,24].

Accordingly, new biomarkers with high diagnostic utility for cardiovascular diseases, including the most common one, hypertension, are constantly being sought [25,26].

**Figure 1:**
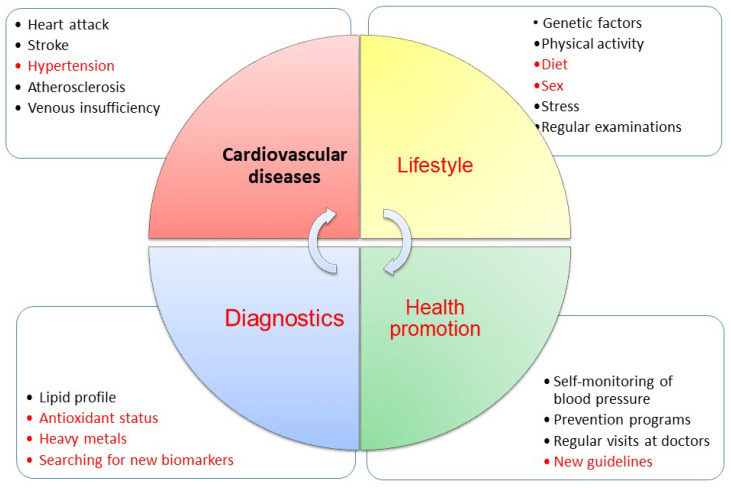
Area of scientific interest.

Only extensive long-term lifestyle monitoring studies can provide data on the population’s health risks of lifestyle diseases. Multi-parameter diagnostic analyses reflecting the latest medical knowledge are essential to achieve this goal. There is an urgent need for fast and reliable CVD diagnosis in the face of the challenge of progressive population ageing. In the era of the rapid development of cardiovascular diseases, it is crucial to identify new biomarkers that will enable early diagnosis, appropriate/personalised treatment, and prevent increased mortality. Only the effective diagnosis, treatment, and monitoring of hypertension can reduce the risk of developing diseases associated with hypertension (Figure 1).

It is important to identify several new parameters of hypertension in men which should be included in CVD diagnostic testing. The latest global guidelines should be used to determine which advanced analytical and diagnostic techniques to apply. The patient’s medical history, including a detailed case history and medical questionnaire, should also be taken into account.

This paper presents research recommendations for future researchers and doctors to improve the diagnosis of hypertension and targeted, personalised treatment. The research proposal includes a lifestyle study, a diagnostic panel with new biomarkers, and an environmental exposure assessment of men working in the metallurgical industry.

## 2. The Sequence of Proposed Studies and Monitoring for Patients at Risk of Hypertension

**Figure 2:**
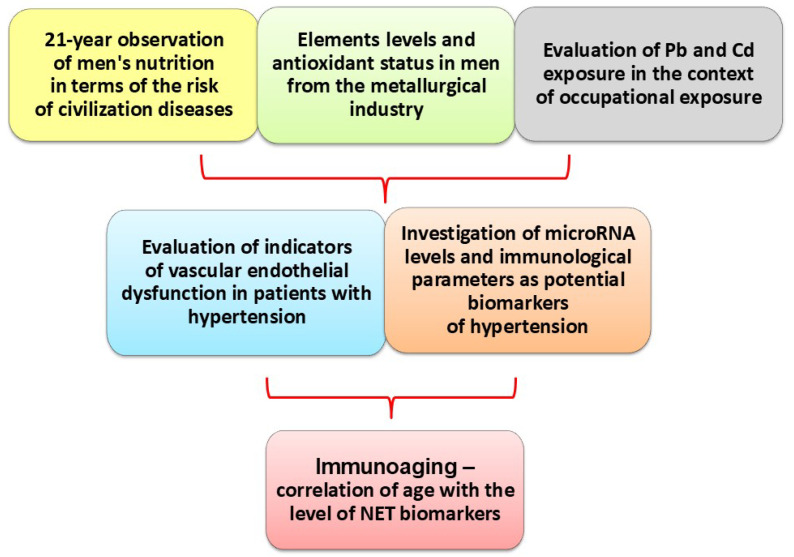
Comprehensive and multidirectional approach to analysing individual parameters, showing study’s vision [6,8,9,10,25,26,27].

Over a few years, I used the following observation (Figure 2) to propose a new diagnostic panel based on a simple design. Each biomarker used in the blood analysis has been carefully selected based on the latest literature, as microRNA is already a well-known recent scientific achievement. Molecular biology is a challenge in both public health and medical care.

This manuscript is divided into sections that evaluate and summarise the research results. Each section is based on my previous research published in other journals, confirming its credibility and usefulness (Table 1). I employed various diagnostic and methodological analyses and selected a population size appropriate to the capabilities of the diagnostic test in question using the indicated methodology. The information on each patient was carefully analysed for future follow-up investigations to provide comprehensive insights into the results. The study group included only men working in the metallurgical industry from the Podlaskie region of Poland, as this was the only opportunity to monitor the same group in one establishment, as I described in a previous paper [6].

## 3. Proposed Comprehensive Research Direction for Researchers and Clinicians

**Figure 3:**
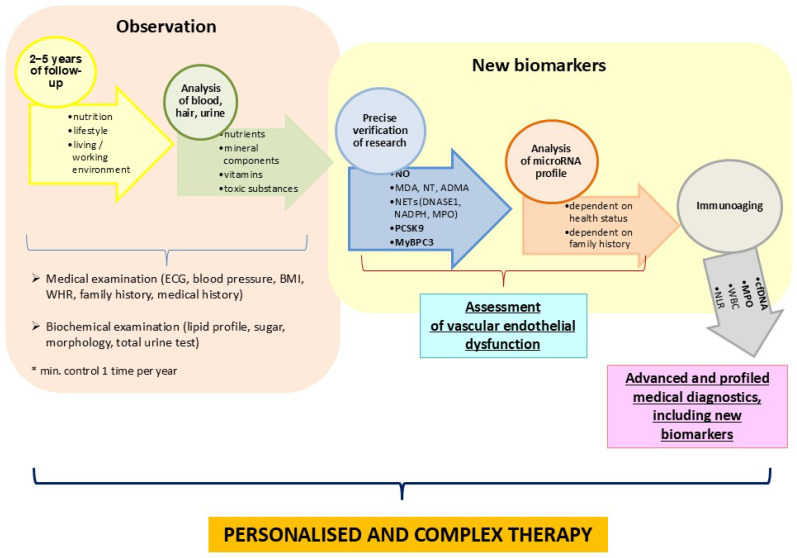
Proposed study and follow-up design.

I propose that this individualised design be included as a research direction and even be considered as a future prevention programme, in this case for hypertension in CVD (Figure 3). The analysis of individual biomarkers should consider several individual parameters, including the patient’s health status, family history, biochemical analysis, haematological tests, and, most importantly, several years of monitoring the patient’s lifestyle, including their place of work and residence. Combining and collecting all this information is the only way to provide a personalised approach to the patient, which is why I emphasise its importance at every visit to the doctor.

I propose a step-by-step management regime and the following possible changes or adjustments to the patient’s treatment (Figure 3):

Stage I. The monitoring of eating habits, including an evaluation of diet (e.g., 24 h dietary history, dietary history of the past three months), lifestyle (physical activity, leisure activities), anthropometric measurements (BMI, WHR, body fat), socioeconomic status, and working conditions. In addition, a physical examination, ECG, blood pressure test, and basic blood tests, including a complete lipid profile.
Stage II. The examination of individual biomarkers (blood, hair, etc.) for vitamins, antioxidant status (e.g., TAS), and minerals (especially Pb and Cd).
Stage III. The analysis of novel blood biomarkers, including NO, PCSK9, MyBPC3, cfDNA, and MPO.
Stage IV. The analysis of selected microRNA molecules that confirm or detect a predisposition to CVDs, including hypertension (e.g., miR-145-5p, miR-1-3p, and miR-423-5p).
Stage V. The verification of a comprehensive and more accurate analysis of the patient’s test results. The application of personalised and targeted treatment.
Stage VI. Follow-up after one year and the potential adjustment of treatment.

In each section, I will present the preliminary results of the studied group of men to explain the purpose of the study in more detail, taking into account the most important and latest findings of other researchers.

It is important to note that Figures below present the methodology used for blood testing in the study group, which often requires advanced knowledge and state-of-the-art technology.

### 3.1. A 21-Year Monitoring of Men’s Diet for Lifestyle Disease Risks

A balanced diet provides the required amount of essential nutrients, minerals, and vitamins [8,18,20,21]. An assessment of the quality of the typical male diet in Poland shows an excessive intake of fat and cholesterol and a lack of minerals and vitamins, contributing to CVD development [19,28].

I used a questionnaire in a prospective study to investigate eating habits and analyse changes in the health of middle-aged men (aged 45 to 75) working in the metallurgical industry [6]. During the history-taking, I collected data on dietary assessment (24 h dietary history, dietary history of the past three months), lifestyle (physical activity, leisure activities), anthropometric measurements (BMI, WHR, body fat), socioeconomic status, and working conditions. The men were evaluated for potential cardiovascular diseases using this information, including a medical history, ECG, blood pressure test, and basic blood tests, such as a complete lipid profile. Therefore, these data should be considered during the first consultation with the doctor, and some could be collected before the visit with the help of a dietician, health educator, or nurse.

A 21-year monitoring [6] of the eating habits of the studied group of men confirmed several unfavourable trends, including a significant increase in BMI, which indicated being overweight. Long-term unfavourable dietary habits significantly increased the study group’s systolic and diastolic blood pressure. Due to their increased atherogenesis in old age and insufficient calcium intake, two risk factors were exacerbated, especially osteoporosis and hypertension. This unique observation confirmed the desirability of following trends in calcium intake in men over an extended period, as its level is an important determinant affecting cardiovascular risk.

As there are no data covering long-term dietary monitoring relating to health status in the same study group, a unique feature of the project was the identical methodology, scope of the study, questionnaire, and assessment of health status determined by the same doctor in the same group of men, at the same time of year, with reference to a study conducted more than 20 years earlier [6]. The comparison of this study’s findings with previous results provided information on the impact of environmental factors and lifestyle on men’s health over the years, which was this study’s greatest and most significant achievement. Therefore, it is also worth using the same methodology in patient follow-up to better monitor their health.

The most important achievement was the confirmation of the validity of the long-term following and monitoring of a population’s diet to determine the risk of lifestyle diseases related to diet, including cardiovascular diseases. This also demonstrated the association of dietary trends with increases in blood pressure and body mass index [6].

Original research [6] has shown that the diet and nutritional status of the men studied require effective interventions to reduce or eliminate their adverse health effects. These interventions can involve health and dietary education programmes for older adults. Consequently, the focus is on the risk of a specific disease and a personalised approach to the patient, making the most of the time of clinicians, dieticians, diagnosticians, therapy, and the patient.

### 3.2. The Evaluation of Trace Elements and Antioxidant Status in Men Working in the Metallurgical Industry

As a continuation of the previous project [6], further investigations were carried out to evaluate the concentrations of selected trace elements and the total antioxidant status (TAS) in relation to the dietary habits of a group of men who were still active in the metallurgical industry [8].

Some elements, such as Zn and Cu, still cause controversy over potential health concerns [8,11]. On the one hand, they are antioxidant micronutrients necessary for proper growth and functioning, while on the other hand, they have undesirable and even toxic effects. Se has similar properties, with a slim tolerance margin between the required and toxic dose. Conversely, Cd and Pb are toxic elements that accumulate in the body and have a half-life of up to 30 years [9,10].

An analysis of the results showed that the average Se concentration in male serum was the only element outside the reference range [8]. Low Se concentrations were also negatively correlated with Pb and Cd concentrations, reducing Se bioavailability. The high recorded TAS in over 40% of the subjects effectively maintains the normal Pb and Cd levels in most male subjects. Similar effects on Pb and Cd are also shown by Zn competing with them for binding sites.

Statistical inference showed no significant correlations between concentrations of the elements studied and the TAS and lifestyle of the men studied, pointing to their work environment as the predominant source of exposure. The very fact that there is a lack of Se in the soil of north-eastern Poland is significant, as it deprives the vegetation of this element, resulting in hyposelenosis.

The most important finding of this work was the demonstration of a positive correlation between Pb and Cd, indicating occupational exposure after excluding lifestyle causes. The results confirmed the need for continuous monitoring of men exposed to heavy metals in the workplace due to their contribution to the development of lifestyle diseases, including CVD. Exposure to heavy metals in Poland remains a significant public health problem, given the increasing occupational and environmental pollution in recent years. Effective preventive action by local authorities should play a key role. Another key achievement was also proving a correlation between Pb and Cd, indicating occupational exposure as a likely factor in reducing Se levels and promoting CVDs and confirming the modulating agonistic effect of Se and TAS on Pb levels [8].

### 3.3. Evaluation of Pb and Cd Exposure as Part of Occupational Exposure

The observations described above [8] prompted me to review the scientific literature for data on the direct and indirect effects of Pb and Cd on human health [9,10]. The presented manuscripts summarise the wide range of physiological, biochemical, and behavioural effects of exposure to toxic elements that are risk factors for CVDs, including hypertension. This is particularly important, as there is strong evidence and data on their toxic effects on health. The symptoms of Pb and Cd poisoning can vary depending on the time of exposure, dose, type of diet, smoking status, age, and health status of those exposed. Ubiquitous environmental exposure to Pb during early foetal life may be a key risk factor for cardiovascular disease throughout life [29]. In addition, another population-based study found an association between chronic Pb exposure and the development of hypertension. Therefore, understanding the impact of the work environment is vital to minimising Pb exposure [30]. The increased blood pressure in the studied group of men working in the metallurgical industry may be caused by the ease of absorption of Pb and Cd. At the same time, the poisoned body, unfortunately, is very slowly cleansed [9,10]. Notably, Cd disrupts endothelial function by decreasing levels of nitric oxide (NO), a known endogenous mediator with vasodilatory effects. Cadmium-induced endothelial dysfunction leads to increased platelet aggregation, proliferation of vascular smooth muscle cells, and thrombophilia [31].

The most important aspect of the publications presented was the compilation of the research results of various authors on the toxic effects of Pb and Cd poisoning. The manuscripts’ added value raised further questions regarding long-term exposure to these elements, especially in terms of damage to the vascular endothelium. The accumulated scientific evidence on the pleiotropic range of dose-dependent toxic effects of Pb and Cd poisoning confirms the desirability of monitoring their concentrations in risk groups [9,10].

In light of the data presented in these articles [9,10], the aim of public policy should be to minimise exposure to toxic metals in the wider human environment and to implement a nationwide programme to monitor their concentrations, especially in occupationally exposed individuals, such as men working in the metallurgical industry.

### 3.4. Evaluation of Vascular Endothelial Dysfunction Indicators in Occupationally at-Risk Men with Hypertension

Vascular endothelial status is of strategic importance for the development of cardiovascular diseases. The metabolically active endothelium plays an important role in regulating vascular tone and permeability, transporting macromolecules, regulating inflammatory responses and coagulation, proliferating the intima, and remodelling the vessel wall [25,32]. A healthy endothelial function determines the maintenance of cardiovascular homeostasis [25].

Endothelial dysfunction, manifested by impaired NO bioavailability in the studied group of men, is a significant risk factor for cardiovascular diseases, including hypertension. Its multi-parameter evaluation of dysfunction is a trend in modern diagnostics. A worsening nitric oxide (NO) deficiency, accompanied by low malondialdehyde (MDA) levels, may be due to an oxidative–antioxidative imbalance. The contribution of NO to regulating blood pressure is indisputable, and it should be among the parameters studied along with the indicators of its action: MDA, NT, and ADMA [33,34,35]. Due to its longer half-life than NO metabolites, NT may help monitor nitric oxide action in vivo [36]. In other studies, high MDA levels in hypertensive patients were accompanied by low levels of antioxidant enzymes [37,38]. Asymmetric dimethylarginine (ADMA), an endogenous competitive NO synthase inhibitor, also appears to be a critical factor in regulating endothelial function. There is evidence to suggest that ADMA is involved in the pathogenesis of spontaneous hypertension [35]. The significantly higher ADMA levels observed in hypertensive patients may be responsible for their reduced NO, as evidenced by the negative correlation of the two parameters. ADMA is formed by the hydrolysis of proteins rich in methylated arginine molecules, mainly histones. In contrast, the increase in histones in circulation is accompanied by the release of NETs, which are also the source of cfDNA. Nowadays, cfDNA is considered a new indicator of vascular endothelial damage, as confirmed by the results of this study, which showed high concentrations of MPO in hypertensive men and a positive correlation between diastolic BP and cfDNA [25].

Circulating free DNA (cfDNA) is a new indicator of vascular endothelial damage in hypertension and acute cardiovascular conditions such as myocardial ischaemia or myocardial infarction [39,40,41]. Neutrophil extracellular traps (NETs) are the source of cfDNA. The first report on NETs described them as a network formed by activated neutrophils. NETosis describes a type of neutrophil death other than apoptosis, resulting in the release of the cell’s genetic material combined with bactericidal proteins into the extracellular space [42]. NETs are one of the body’s primary non-specific defence strategies against pathogens. However, the imbalance between the persistence of NETs and their effective degradation leads to the development of low-grade chronic inflammation and vascular damage, which is a risk factor for CVDs. In addition, NETs can induce autoimmune processes due to the easy access of lymphocytes to autoantigens present in the network structures of the circulatory system, mainly histone proteins and MPO [43]. The evaluation of initial NET formation and the presence of biomarkers, MPO, anti-MPO autoantibodies, or cfDNA activity is currently a diagnostic goal for many lifestyle diseases, including CVDs [25].

Therefore, the next stage of this research was to identify the most recent biomarkers of endothelial dysfunction among several key parameters that play an important role in vascular endothelial function in a study group of hypertensive subjects [25].

**Figure 4:**
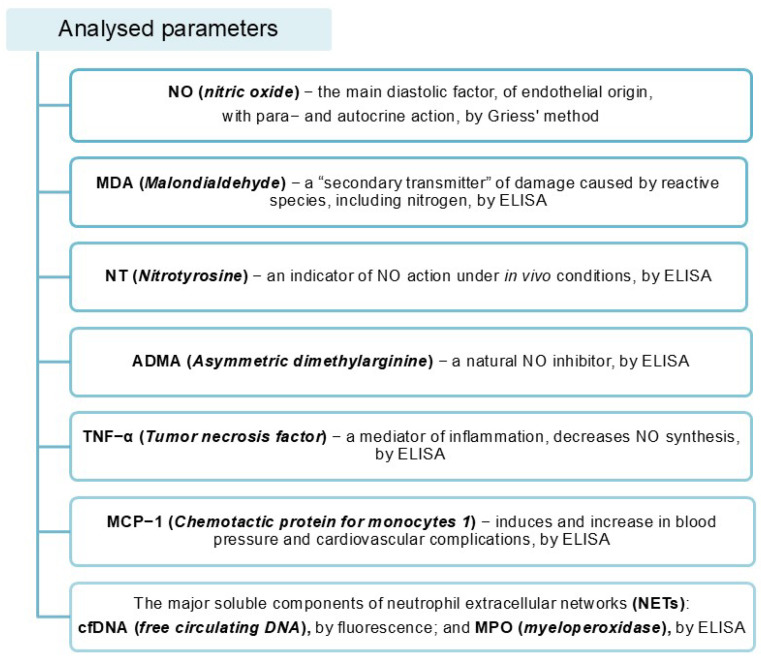
Parameters analysed [25].

In a comprehensive approach to the experiment, the concentration of NO, the main endothelium-derived diastolic factor with para- and autocrine effects, was examined. The amount of MDA, a secondary transmitter of damage caused, among other things, by reactive nitrogen species, was determined. The concentrations of NT, an indicator of NO activity in vivo, and the amount of ADMA, a natural NO inhibitor, were also assessed. In addition, the levels of the highly pro-inflammatory tumour necrosis factor (TNF-α) and monocyte chemoattractant protein (MCP-1), which are involved in damage to the vascular endothelium and the formation of atherosclerotic plaques, were determined (Figure 4). The novelty of the study was in evaluating the levels of the main soluble components of neutrophil extracellular traps: cfDNA and MPO. Recent scientific reports confirm their involvement in endothelial damage [25].

An extremely useful aspect of this work was its demonstration of various mechanisms involved in the development of hypertension and other cardiovascular disorders with a common NO component. The high levels of MDA, NT, and ADMA in hypertensive men support a predominant role of oxidative stress in endothelial dysfunction. In turn, the analysis of results concerning TNF-α and MCP-1, the main mediators of inflammation, in patients with hypertension and other accompanying cardiovascular disorders indicates the dominance of inflammation in the course of these complications. It is likely that the low-grade inflammation associated with the release of pro-inflammatory proteins and the development of target organ damage that characterises hypertension results from NETs in the vascular system [25].

The observed increases in blood pressure, lipid and carbohydrate metabolism disorders, body weight, and age [6] have a common denominator in endothelial dysfunction [25]. The negative correlation of age and NO in the control group indicates a progressive reduction in nitric oxide levels in the body. Eliminating risk factors from the diet, especially simple carbohydrates and saturated fatty acids, and thus their negative impact on endothelial function, offers the potential for appropriate and targeted prevention in middle-aged men (Figure 3).

The most significant achievement of this publication was the proposal of a new diagnostic profile based on the distribution of the results of selected parameters, including the examination of NO and cfDNA concentration, in the risk assessment and/or diagnosis of endothelial dysfunction in hypertensive men [25].

### 3.5. Investigation of MicroRNA Levels and Immunological Parameters as Potential Biomarkers for Hypertension in High-Risk Male Patients

There is high hope for the use of mRNA therapy in CVD [26]. The preliminary results from preclinical phase studies on the use of mRNA therapy in myocardial ischaemia are promising [44,45]. However, targeted mRNA therapy must be preceded by studies evaluating the levels of microRNA molecules, i.e., the potential points of interest. MicroRNAs are small regulatory RNAs involved in post-transcriptional gene silencing [46,47]. The primary function of microRNAs, through interactions with mRNAs, is regulating genes crucial for the development and functioning of the body, such as cell division, cell differentiation, apoptosis, blood vessel formation, and tumour formation [48].

Several microRNAs have been shown to be involved in heart disease, e.g., low plasma levels of miR-145-5p are found in patients with acute myocardial infarction. In contrast, high levels are found in patients with a genetic predisposition to dilated cardiomyopathy. MiR-1-3p has been proven to play a key role in the development and physiology of muscle tissue, including the heart. Changes in its expression lead to hypertrophy, myocardial infarction, or arrhythmias [48,49]. MiR-423-5p was initially identified as a circulating biomarker of heart disease [50]. Research into the potential use of microRNAs in medicine is highly promising. Still, even at this stage, it has been suggested that diagnostic panels for specific diseases should be developed, as the evaluation of individual microRNAs is insufficient.

Profiling microRNAs, short non-coding single-stranded RNAs, shows great potential. The main function of miRNAs, through their interaction with mRNAs, is to regulate 30% of genes in the human genome that are crucial for the normal development and function of the organism [26].

However, in recent years, the “myocardial infarction risk gene” has been identified as the MYBPC3 gene for myosin-binding protein C (MyBPC3), which regulates the function of the heart muscle and protects it from proteolysis. MyBPC3 is rapidly released into the blood after myocardial injury and thereby may be a biomarker for the early stages of myocardial infarction [51,52]. It is estimated that 1 in 100 people worldwide carries the MYBPC3 gene mutation, which may be accompanied by reduced miR-155 expression [53,54]. Recent studies have shown that cholesterol loading reprogrammes the microRNA-143/145–myocardin axis, changing the phenotype of aortic smooth muscle cells to a dysfunctional one [55,56]. These findings have intensified efforts to search for the cause of impaired LDL cholesterol metabolism [55,57].

**Figure 5:**
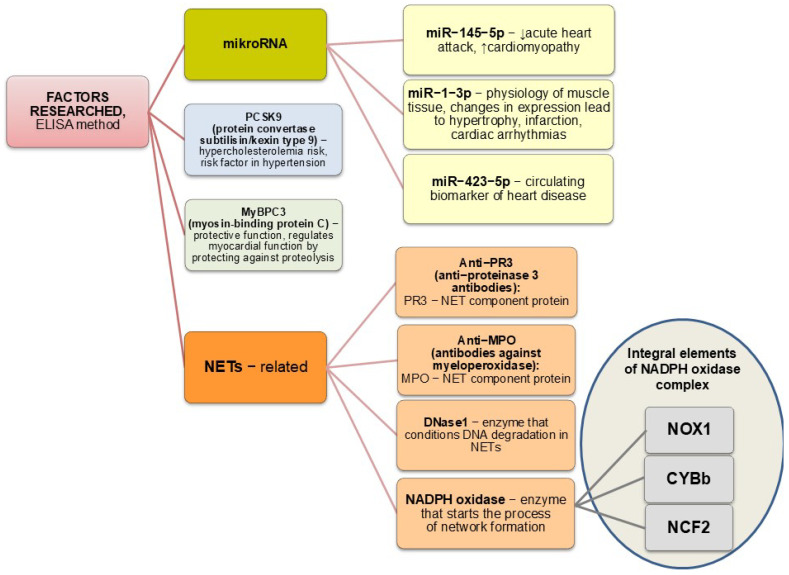
Parameters analysed [26].

Given the proven role of several microRNAs (miR-145-5p, miR-1-3p, miR-423-5p) in heart disease, I decided to include them in my panel studies (Figure 5) of hypertensive men [26].

In my pioneering study, high concentrations of miR-145-5p, miR-1-3p, and miR-423-5p were observed in the sera of male patients, indicating the involvement of selected microRNAs in hypertension [26]. According to the literature, miR-145-5p negatively correlates with TNF-α. High levels of miR-145-5p likely inhibit the inflammatory response through the TNF-dependent pathway, confirming the lack of significant differences in the levels of this cytokine in hypertensive men [25]. Studies on the potential use of microRNAs in medicine have great potential, but they need to be validated in a large cohort, taking into account all individual and methodological variables. The positive correlations between the microRNAs evaluated in my study indicated a significant relationship between them, which confirmed the validity of this panel.

Among the proteins with a proven role in the development of cardiovascular diseases, my study focused on proprotein convertase subtilisin/kexin type 9 (PCSK9), investigating the causes of the changes in total cholesterol and its fractions in the male subjects identified in the previous study [6]. In contrast to the negative role of PCSK9, MyBPC3 has a protective function, regulating myocardial function and protecting it from proteolysis, which is why it was included in this project [26].

Literature data indicate that PCSK9 binds to LDL receptors, promoting their degradation. This results in a reduced rate of LDL cholesterol removal from plasma and a higher risk of hypercholesterolaemia [58]. The latest therapeutic strategies attempt to utilise specific microRNAs, which can be targeted and, thus, regulate PCSK9 expression [59,60].

The observed high levels of PCSK9 in hypertensive men may have been a direct cause of the high levels of total cholesterol and LDL cholesterol in these patients. The observation of the highest levels of PCSK9 in men diagnosed with hypertension at the same time as the highest levels of total cholesterol and LDL cholesterol in this group indicates its potential role as an early biomarker of hypertension. The positive correlation between PCSK9 and MyBPC3 in patients with diagnosed hypertension and the similar direction of changes in the levels of these proteins in other groups of men indicate a significant association between them. The highest levels of MyBPC3 in hypertensive men suggest a potential mechanism that protects the myocardium from the consequences of chronic hypertension [26].

The interesting results of this innovative research, described in a previous paper [25], on the evaluation of factors that may cause vascular endothelial dysfunction in hypertensive men, showed a link between the disease and the level of biomarkers of neutrophil extracellular trap (NET) formation, prompting me to investigate this phenomenon further.

On the one hand, NETs protect against pathogens. Still, on the other hand, they can cause the production of antineutrophil cytoplasmic antibodies (ANCAs), which target cytoplasmic neutrophil antigens, including the following: the cytoplasmic ANCA (c-ANCA) against proteinase 3 (anti-PR3) and peripheral ANCA (p-ANCA) against myeloperoxidase (anti-MPO). Also referring to an earlier study [25], my study evaluated the levels of anti-PR3 and anti-MPO antibodies in the studied group of men. In contrast, NADPH oxidase and DNase I are two strategic enzymes in NETosis, which closed the list of parameters studied. The proteins nicotinamide adenine dinucleotide phosphate oxidase 1 (NOX1), cytochrome b-245 beta polypeptide (CYBb, NOX2), and neutrophil cytosolic factor 2 (NCF2, NOXA2) form an integral part of the NADPH oxidase complex, which activates NET formation. DNase I conditions the efficient DNA degradation of NETs in the body, participating in maintaining normal blood circulation [26].

The elevated DNase I levels in the men may suggest that the enzyme has a protective effect against the effects of NETs in the vascular system. However, the high negative correlation between DNase I and patient age indicates that this protective mechanism may be impaired in older men [26].

The high negative correlation between age and NOX1 may be related to impaired neutrophil function in non-specific killing mechanisms associated with NADPH oxidase activity in the elderly. The low levels of NOX1 and CYBb found in all the hypertensive men may be due to ageing or the “wear and tear” of oxidase subunits in NET formation. Their levels of NOX1 and CYBb may also be due to high amounts of microRNAs [26].

I hypothesised that the activation of autoimmune processes may accompany the formation of NETs in hypertensive men due to the easy access of lymphocytes to the autoantigens present in NET structures in the circulatory system. The negative results of anti-MPO and anti-PR3 antibodies in all the groups of studied men exclude the development of NET-related autoimmune processes in the course of hypertension [26].

The key findings of this experiment show that the focus should not be on identifying individual proteins or microRNA molecules but rather on establishing a profile of biomarkers for cardiovascular disease, which should include PCSK9 and MyBPC3 as they have the greatest diagnostic potential for male hypertension [26].

Changes in heart disease markers are already evident in the course of hypertension, which sets the direction for future CVD-focused projects. Only the effective diagnosis, treatment, and monitoring of blood pressure can reduce the risk of developing CVD. There is an urgent need for fast and reliable diagnostics of cardiovascular diseases in the face of the challenge of a rapidly ageing population.

### 3.6. Immunoageing—The Correlation Between Age and NET Biomarkers, the First Line of Immune Defence in a Pathological Condition

Further strategies and prospective studies need to address the problem of an ageing population. Ageing is closely related to the ageing of the immune system. Most scientific evidence seems to support ageing theories such as multiple damage accumulation and irreversible changes at the molecular level within the cell [27]. On the other hand, the immunological theory of ageing centres around impaired immune memory. Impaired leucocyte function results in the ineffective killing of pathogens on the one hand and the treating of one’s own cells as foreign on the other, which initiates self-destructive processes [28]. The demonstrated changes and disorders based on the long-term monitoring of the study group should always be reviewed in the context of the physiologically progressive changes accompanying ageing.

The negative correlation between DNase I and men’s age, as demonstrated in the above-mentioned study [26], justified further analyses evaluating the impact of ageing on the formation of extracellular neutrophil traps in men [27].

A typical feature of the immune system is the quantitative and functional variability of its components, with neutrophils playing a special role in maintaining homeostasis. They form the core of the innate immune response and support the acquired immune response. Studies show that the immune system of newborns and children is immature, while maturity corresponds to the immune system reaching its full potential. Conversely, inevitable ageing is associated with immune dysfunctions of cellular and humoral responses [27].

**Figure 6:**
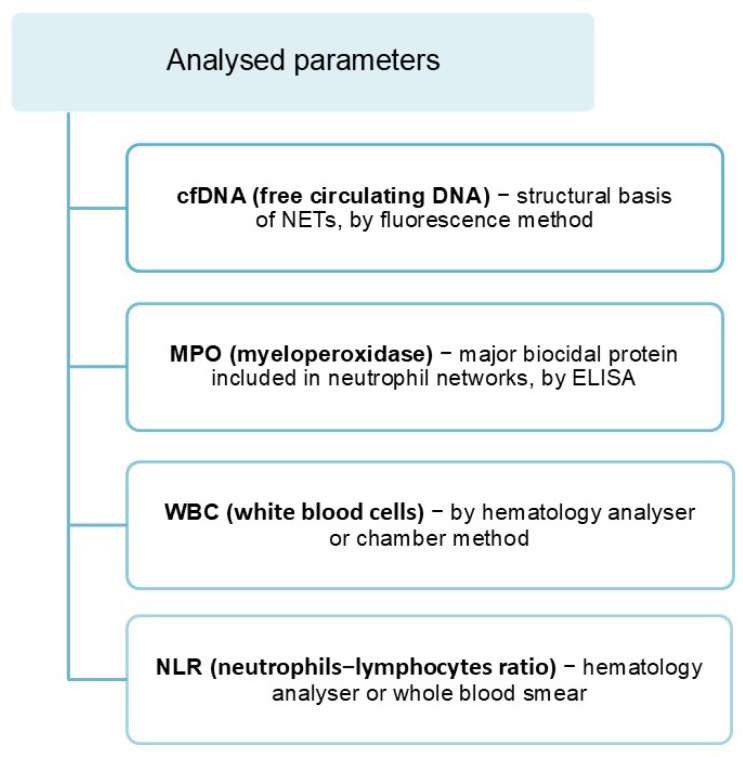
Parameters analysed [27].

Considering the above information and the lack of data on changes in NET formation with age, my pilot project aimed to evaluate the main biomarkers released from NETs in the whole blood sera of men of different age groups. The concentrations of cfDNA, the structural basis of NETs, and myeloperoxidase (MPO), the main biocide protein contained in NETs, were determined (Figure 6). In addition, the neutrophil-to-lymphocyte ratio (NLR) was determined.

Finding a positive correlation between MPO and cfDNA concentrations, confirming the close relationship between the parameters studied and the release of NETs into the extracellular space, was crucial to achieving the project’s objectives [27].

The low MPO and cfDNA levels in the serum of older men indicated disturbances in NET formation in this age group. This study showed that the NLR tended to increase with age, confirming the shift in the leucocyte population to neutrophils. The negative correlation between the neutrophil and lymphocyte counts and the correlation between the NLR and lymphocytes supported this conclusion [27].

This study’s important findings were the low levels of MPO and cfDNA in older individuals, which indicated impaired NET formation and were likely a cause of the impaired response to pathogens. The comparable MPO and cfDNA levels in the analysed groups can be explained by distinct mechanisms, such as the immature immune system during puberty and its impairment in the elderly. The resulting data provided valuable information on pathogen elimination via NETs while indicating the need to support non-specific responses in the elderly [27].

The key accomplishment of this study was demonstrating a gradual increase in MPO and cfDNA with age and comparable amounts of NET biomarkers in adult males, which showed that NET production was functioning properly and suggested that neutrophil maturity enabling NET release is achieved during adolescence.

Another significant contribution was the understanding of the ontogenesis of the immune system in terms of NET formation based on the parabolic distribution of MPO and cfDNA concentrations depending on age, indicating the need to support the innate response in the elderly.

## 4. Study Limitations

This comprehensive result analysis introduces a novel line of research but requires confirmation in a bigger study group. The observations were limited by the cost of analyses and the inability to re-invite patients due to the shutdown of the metallurgical plant. I was also unable to carry out the annual follow-ups suggested in Figure 3. Unfortunately, this was due to the group’s characteristics, ageing, and lack of financial means. Even the suggested panel studies can often only be carried out using highly advanced techniques currently available in research laboratories but not for commercial testing.

## 5. Suggestions for Future Research Directions

The strength of this study is its multi-faceted approach to future preventive testing in men. However, exposure to other heavy metals (e.g., cadmium, mercury, nickel) or bisphenol and its derivatives are also worth including in the study. In this case, urine, blood, hair, and nail analyses should be carried out to show the effects of short- and long-term exposure to these substances.

This preventive regimen could also be applied to women, but, in this case, attention should be paid to hormonal changes during pregnancy, lactation, or menopause, which are often more pronounced than in men.

## 6. Conclusions

In the era of the rapid increase in lifestyle diseases related to the cardiovascular system, new biomarkers are being sought for early diagnosis and appropriate/personalised therapy to prevent rising death rates. The new scientific evidence from basic research strongly confirms the advisability of using it to prepare a diagnostic panel. This proposed study and follow-up design will definitely improve the detection and monitoring of hypertension in men.

The analysed parameters play a significant role in prevention, so several new parameters (NO, cfDNA, MPO, PCSK9, MyBPC3, miRNA, TAS, Pb, Cd) with prognostic and/or predictive potential should be included in screening tests, along with the direction of genetic testing, which seems to be at the leading edge of the state-of-the-art methods for assessing vascular endothelial dysfunction, right after NO assessment. This study also confirms the necessity of extensive screening in middle-aged men by healthcare professionals for the risk of developing hypertension.

Undoubtedly, these findings prove that the analysed parameters are clinically relevant to the progression of hypertension, which will guide future projects focused on cardiovascular disorders.

The latest basic research findings should be used to develop up-to-date prevention programmes at both local and regional levels, taking into account places of work and residence.

## Figures and Tables

**Table 1:** My comprehensive health assessment included the following biological material and literature data.

No.	A Comprehensive Health Assessment, Published and Described in the Manuscripts Below	References
1.	The changes in diet and lipid metabolism in a 21-year prospective study of men working in the metallurgical industry in north-eastern Poland and the changes in their health status associated with cardiovascular risk factors.	[6]
2.	The concentrations of selected trace elements and total antioxidant status in the studied group of men as likely CVD contributors, along with the group’s dietary habits.	[8]
3.	The available data on Pb and Cd exposure’s effects on different age groups.	[9,10]
4.	A profile of new biomarkers of endothelial dysfunction among parameters playing a key role in vascular endothelial function in male hypertension.	[25]
5.	The diagnostic potential of microRNA profiling and immunological parameters associated with cardiovascular dysfunction in male hypertension.	[26]
6.	The impact of ageing on neutrophil extracellular trap (NET) formation in men.	[27]
